# Prevalence of Obesity and Associated Dietary Habits among Medical Students at King Khalid University, Southwestern Saudi Arabia

**DOI:** 10.3390/medicina60030347

**Published:** 2024-02-20

**Authors:** Ahmed A. Mahfouz, Safar Abadi Alsaleem, Mohammed Abadi Alsaleem, Ramy Mohamed Ghazy

**Affiliations:** 1 Department of Family and Community Medicine, College of Medicine, King Khalid University, Abha 61421, Saudi Arabia; asalslim@kku.edu.sa (S.A.A.); ma-bade@kku.edu.sa (M.A.A.); ramy_ghazy@alexu.edu.edu (R.M.G.); 2 Department of Epidemiology, High Institute of Public Health, Alexandria University, Alexandria 61421, Egypt; 3 Tropical Health Department, High Institute of Public Health, Alexandria University, Alexandria 61421, Egypt

**Keywords:** healthy eating, obesity prevalence, dietary habits, medical students, gender differences, nutritional counseling

## Abstract

*Background and Objectives*: Healthy eating is a crucial approach to improving overall health, encompassing a well-balanced diet of natural and fresh foods, plenty of fruits and vegetables, and foods rich in minerals and vitamins. This study aimed to assess the prevalence of obesity and associated dietary habits among medical students at King Khalid University, Aseer, Saudi Arabia. *Materials and Methods*: This observational cross-sectional study was conducted through face-to-face interviews. A structured predesigned questionnaire was used to collect data. *Results*: A total of 540 medical students were included; 43.3% of participants were aged 20–22 years, 24.8% were in the 3rd year, and 82.0% had an average income level. Of them, 21.9% were overweight and 14.6% were obese. There was a significant association between obesity and grade (*p* = 0.004). Significant differences were observed between males and females in adding sugar to beverages, the frequency of eating out, cooking meat, and drinking water (*p* < 0.05). The predictors of obesity were being male (OR = 3.5, 95% CI [1.6–7.8], *p* = 0.002), age (OR = 1.8, 95% CI [1.1–3.0, *p* = 0.019], being at grade 2 (OR = 38.8, 95% CI [4.0–375.8], *p* = 0.002), having grilled meat (OR = 0.42, 95% CI, [0.20–0.99], *p* = 0.048), using artificial sweeteners [OR = 0.24, 95% CI [0.08–0.73], *p* = 0.012], and drinking sparkling bottled water (OR 8.6, 95% CI [1.2 333–63.8], *p* = 0.034). *Conclusions*: The study revealed a high prevalence of obesity and overweight among medical students of both sexes. It recommends education on healthy eating habits, balanced nutrition, and regular physical activity, as well as gender-specific health initiatives, nutritional counseling, and the inclusion of physical activity.

## 1. Introduction

Noncommunicable diseases (NCDs) are the leading causes of death and disability in developed and developing countries and include conditions such as cardiovascular disease, cancer, chronic respiratory diseases, diabetes, obesity, and cognitive impairment [1]. While genetic and environmental risk factors for NCDs are well known, there is a strong emphasis on modifiable elements related to lifestyle at the individual level [2,3,4]. Dietary choices influence the risk of conditions such as hypertension, hypercholesterolemia, overweight/obesity, and inflammation. As a result of these factors, the likelihood of diseases with significant morbidity and mortality, such as cardiovascular disease, diabetes, and cancer, increases [2].

A well-balanced diet of natural and fresh foods, lots of fruits and vegetables, and foods high in minerals and vitamins is essential to improve overall health [5]. It involves developing consistent behaviors and dietary habits that promote and sustain physical and psychological well-being. The dynamics of healthy eating are influenced by a wide range of individual and collective factors, including social and environmental factors [6]. In fact, eating behavior is defined as the usual actions and choices people make regarding their food consumption, including the selection of foods, culinary preparations, and the quantities consumed [7].

Due to a rapid increase in socioeconomic status at both the government and population levels, the trajectory of eating patterns in Saudi Arabia has undergone significant changes in recent decades, affecting various age groups, particularly children and youth [8]. Previous research has found that Saudis consume more refined foods and animal products than they do fruits and vegetables [9,10]. In a recently published systematic review, the highest reported prevalence of obesity among large Saudi studies (sample size > 10,000) was 35.6%. The country’s rising obesity rate is expected to result in 2.26 million new cases of type 2 diabetes, liver disease, and liver cancer by 2040. According to projections, a 20% reduction in obesity prevalence could prevent over 150,000 deaths by 2050 [11].

Indeed, university student life is marked by numerous shifts in eating behaviors and dietary patterns [12,13]. Students who move from high schools to universities struggle to maintain healthy eating habits, a struggle that is often attributed to time constraints and stressors, resulting in behaviors such as eating out without meals, unhealthy snacking, eating out, and fast food consumption [14]. There is a knowledge gap about healthy dietary options, which has the potential to passively shape eating habits and nutrition [15]. Interestingly, the level of knowledge was found to be significantly associated with dietary habits [14]. Due to their extensive medical knowledge of healthy eating patterns, medical students are expected to serve as role models for their peers. Although they have adequate knowledge, medical students struggle to apply it, a phenomenon attributed to the stressors of university life and medical studies [15,16].

Given the observed shifts in eating behaviors among university students globally and considering Saudi Arabia’s unique socioeconomic changes, we propose that medical students at King Khalid University exhibit distinctive dietary patterns. We anticipate a high prevalence of obesity and unhealthy eating habits, such as meal skipping, unhealthy snacking, and reliance on fast food, particularly among those transitioning from secondary schools to universities. This study aimed to provide valuable information on the dietary behaviors of medical students in Saudi Arabia. Furthermore, our goal was to address the prevalence of overweight and obesity among this specific group. By doing so, we aimed to establish a foundation for targeted interventions that promote healthier eating habits and help prevent the associated risks of NCDs.

## 2. Materials and Methods

### 2.1. Study Design

This was an observational cross-sectional study. Face-to-face interviews were used as the primary method of data collection from 15 August to 14 September 2024.

### 2.2. Study Population

This study focused on medical students enrolled at King Khalid University, Abha, Saudi Arabia, with a total of 600 students spanning grades 1 through 6. Initially, a pilot questionnaire was administered to a sample of 30 grade 1 students. Subsequently, all students in grades 2 to 6 were invited to participate in the study; the response rate was 83%.

### 2.3. Variables and Measurements

This study’s variables included demographic, dietary, and lifestyle factors. The participants’ demographics included age, gender, academic year, household size, and income level. Dietary habits were investigated using variables such as daily meal frequency, meal consistency, snacking patterns, milk and dairy product preferences, and participants’ eating out frequency.

### 2.4. Questionnaire Design

The questionnaire consisted of three sections:

In the first section, participants were categorized according to their age into three groups: 18–19 years, 20–22 years, and above 22 years. They were then categorized by gender (male or female), academic year (2nd–6th year), income level, (below average, average, or above average), and household size, which was categorized into below 3 members, 3–6 members, or above 6 members.

The second section: respondents were asked to indicate their typical daily meal frequency, with the following options: 2 meals to 5 or more meals. Participants were then prompted to share whether they consume meals at regular times, providing choices of “no”, “yes, but only some of them”, and “yes, all of them”. The survey also explored snacking habits, allowing respondents to specify how often they snack between meals, ranging from “never” to “few times a day”. Furthermore, the type of milk and dairy beverages consumed was addressed, offering choices such as “full fat”, “low fat”, and “no fat (skimmed)”. Participants were asked about their preferences for sugar in hot beverages and the use of artificial sweeteners. They were also asked about their addition of salt to prepared meals, with options indicating frequency. Finally, participants were asked about their frequency of eating out, providing a spectrum from “never” to “few times a day”.

The third section: During the study, the participants were weighed, and their height was measured. The height and weight measurements of the medical students were taken using standardized methods. A stadiometer was used to measure height, and participants stood barefoot against the wall, ensuring that their heels, buttocks, shoulders and back of their head were in contact with it. Their height was measured to the nearest centimeter. Scales were used to measure weight. Participants walked on the scale barefoot or in minimal clothing, making sure they did not wear heavy outerwear or accessories that could interfere with the accuracy of the measurements. Their weights were measured to the nearest kilogram. The equipment used for height and weight measurements was consistent among all participants, ensuring that the data collected was accurate and consistent. Measurements were taken under controlled conditions to minimize variables that could affect the accuracy of the measurement.

To calculate the body mass index (BMI), the body weight in kilograms was divided by the height squared in meters. A BMI below 18.5 is categorized within the underweight range. A BMI ranging from 18.5 to less than 25 designates a healthy weight range. A BMI falling between 25.0 and less than 30 is placed within the overweight category. A BMI of 30.0 or higher denotes the obesity range [17].

### 2.5. Pilot Testing

The questionnaire was pilot tested with a small group of first-year medical students to assess its clarity, relevance, completeness, and the time required for participants to respond. The purpose of this preliminary test was to identify any potential problems or ambiguities in the questionnaire design. Feedback from the pilot study was carefully analyzed and changes were made to improve the readability of the questionnaire based on the feedback. This iterative process ensured that the final version of the questionnaire was well designed, user-friendly, and able to capture all the information required for the study. Data collected during the pilot study was excluded from the final data analysis.

### 2.6. Data Collection

Researchers engaged with the participants on campus and provided them with information regarding the study’s objectives.

### 2.7. Face-to-Face Interviews

The principal investigator conducted a comprehensive training session for a group of five final-year medical students, equipping them with the necessary skills and knowledge for data collection. Subsequently, these trained interviewers were responsible for conducting face-to-face interviews with the participants in private settings on campus, prioritizing confidentiality, and privacy. During these interviews, the participants were guided to respond to the questionnaire items, allowing for a direct and personal interaction that facilitated a comprehensive understanding of the responses.

### 2.8. Study Outcomes

This study focused on assessing the prevalence of overweight and obesity among medical students at King Khalid University while also shedding light on their eating habits.

### 2.9. Statistical Analysis

All data collected were numerically coded, processed with Microsoft Excel 2016, and analyzed with statistical software (e.g., SPSS v 27). To summarize the study variables, descriptive statistics were used, with percentages and frequencies used for categorical data and mean and standard deviation used for numerical data. The association between categorical variables was evaluated using the Chi-square test. Furthermore, binary logistic regression was used to identify the main determinants of obesity among students, considering factors such as diet habits. Statistical significance was set at *p* < 0.05.

### 2.10. Ethical Considerations

Participants were fully aware of the research objectives prior to the start of the study. Each participant was then asked to provide written informed consent. The study was approved by the King Khalid University Research Ethics Committee. The Helsinki Declaration’s tenets were strictly followed throughout the research process. The study objectives were clearly communicated to the participants prior to the start of the study. This entailed giving a comprehensive overview of the research’s objectives. After the screening for obesity and overweight, individuals diagnosed with these conditions received educational messages emphasizing the importance of maintaining a healthy weight and adopting a nutritious diet. Subsequently, they underwent further investigations to assess other systems and potential health issues.

## 3. Results

### 3.1. Sociodemographic Characteristics

Table 1 provides demographic information on a group of individuals based on age, sex, academic year, income level, and household size. A total of 540 participants were included in this study. Their mean age was 21.7 ± 1.5 years. More than three-fifths of the individuals fell into the age category of 20–22 years, making up 43.3% of the sample. There was a slightly higher representation of males (53.3%) compared to females (46.7%). The distribution across academic years was relatively even, with the highest representation in the 3rd year (24.8%) and the lowest in the 6th year (10.6%). Most individuals had an average income level (82.0%), while only a small percentage fell below average (1.3%) or above average (16.5%). The mean household number was 6.6 ± 2.8 members. More than half of the individuals came from households with more than six members (51.7%), followed by households with three to six members (38.5%), and households with less than three members (9.8%). 

### 3.2. Distribution of the Study Participants according to the Body Mass Index

**Figure 1:**
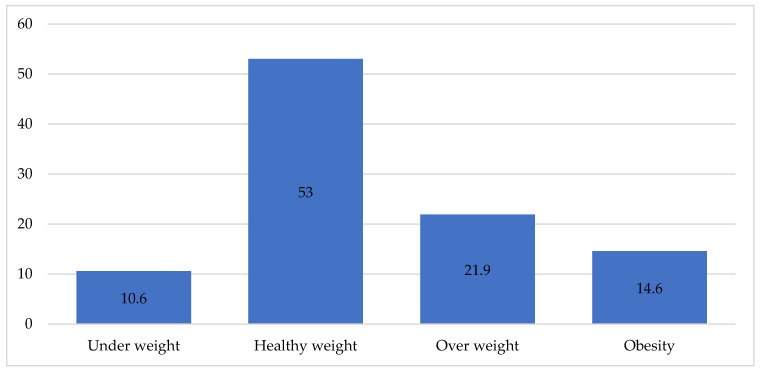
The distribution of medical students at King Khalid University according to their body mass index.

More than half of the medical students fell within the “normal weight” category, constituting 53% (n = 286 students). A notable portion of the students was classified as “overweight”, comprising 21.9% (118 students). Students classified as “obese” made up 14.6% (79 students) of the population. A smaller proportion fell into the category of “underweight”, accounting for 10.6% (57 students). Figure 1.

**Figure 2:**
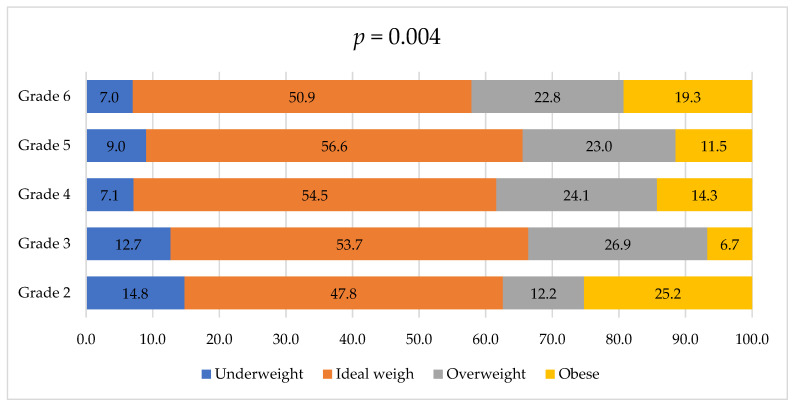
Distribution of medical students based on their weight across different university grades.

Figure 2 illustrates the distribution of students across different weight categories (underweight, normal, overweight, obese) in grades 2 through 6. In grade 2, approximately 14.8% of the students were underweight, 47.8% were of normal weight, 12.2% were overweight, and 25.2% fell into the obese category. Grade 3 shows a slightly different pattern, with 12.7% underweight, 53.7% at normal weight, 26.9% overweight and 6.7% classified as obese. In Grade 4, 7.1% were underweight, 54.5% at normal weight, 24.1% overweight, and 14.3% obese. In grade 5, 9.0% were underweight, 56.56% at an ideal weight, 22.95% overweight, and 11.48% obese. Lastly, in grade 6, 7.0% were underweight, 50.9% at normal weight, 22.8% overweight, and 19.3% obese. These differences between different grades were statistically significant (*p* = 0.004).

### 3.3. Dietary Habits of the Medical Student

Table 2 presents the findings of a survey on various aspects of individuals’ dietary habits and eating behaviors. Approximately two-fifths of the respondents (40.9%) usually consumed three meals, and 13.5% reported having all their meals at regular times. Only a small percentage reported never snacking (4.3%), 63.0% preferred full-fat milk and dairy beverages, 39.4% added one teaspoon of sugar (or honey) to their hot beverages, a smaller percentage (7.8%) used artificial sweeteners, and 43.5% reported adding salt to most of their meals. Eating out was a common practice, with 34.1% doing so a few times a week, followed by once a week (25.7%) and once a day (10%). A small percentage (3.7%) reported never eating out.

### 3.4. Variation in Dietary Patterns Based on Gender among Medical Students

Table 2 compares the dietary habits between male and female medical students at King Khalid University. There were no significant differences between male and female students in terms of the number of meals they usually consume daily (*p* = 0.922). Both sexes report predominantly consuming two or three meals per day. Similarly, there was no significant difference in the distribution of students who consume meals at regular times. Although there were no significant differences in the type of milk and dairy beverages consumed overall, a slightly higher percentage of male students reported consuming full-fat options (*p* = 0.065). Statistically significant differences exist in the frequency of snacking between male and female students (*p* = 0.001). A higher percentage of male students report never snacking between meals, while a higher percentage of female students snack more frequently, including once a day and a few times a day. No significant differences were found in the frequency of adding salt to meals and sandwiches between male and female students. Significant difference was observed in adding sugar to hot beverages, with a higher percentage of male students not adding sugar and a higher percentage of female students adding one teaspoon of sugar and using artificial sweeteners (*p* = 0.001). A significant difference was observed in the frequency of eating out (*p* = 0.001). Male students tend to eat out more frequently, with higher percentages across all categories, especially in the “few times a week” and “once a day” categories. Statistically significant differences were observed across sexes concerning the preparation of meat and the drinking of water (*p* = 0.001).

**Figure 3:**
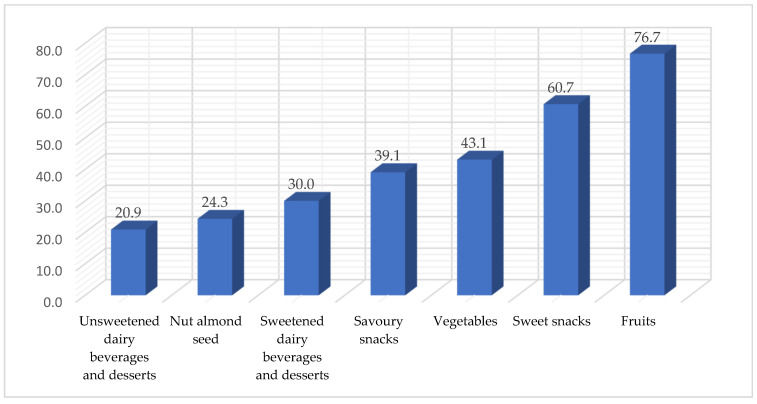
Types of food usually consumed between meals during the week by medical students.

Notably, a substantial number of medical students, 76.7%, opted for fruits as a between-meal snack. Additionally, sweet snacks were popular, with 60.7% of students choosing them. Vegetables (43.1%) and savory snacks (39.1%) also made up a significant portion of between-meal choices. Almond nut seeds, at 24.3%, indicated a moderate but noteworthy preference for nutty and protein-rich snacks. On the other hand, sweetened dairy beverages and desserts (30.0%) and unsweetened dairy beverages and desserts (20.9%) were chosen by a relatively smaller percentage of students. Figure 3.

Table 3 presents the various variables studied in relation to obesity, including age, sex, academic year, household size, financial situation, eating habits, and preferences. The mean age for the non-obese and obese individuals was quite close, (*p* = 0.724). There was a significant association between sex and obesity (*p* < 0.001). Almost one fifth of the males were obese (21.9%), while a smaller percentage of females were obese (6.3%). The academic year seems to be significantly associated with obesity (*p* < 0.001). There was no significant association between household size and obesity (*p* = 0.952). The students’ financial situation did not appear to be significantly associated with obesity (*p* = 0.358). The number of meals consumed daily, and the regularity of meal consumption did not show significant associations with obesity (*p* > 0.05). The frequency of snacking between meals was not significantly associated with obesity (*p* = 0.444). There was a marginal association between adding sugar to hot beverages and obesity (*p* = 0.08). Similarly, the use of artificial sweeteners was significantly associated with obesity (*p* = 0.027). Both the addition of salt to meals and sandwiches and the frequency of doing so were significantly associated with obesity (*p* = 0.028). There was a significant association between consuming sparkling bottled water and obesity (*p* = 0.048). The frequency of eating out was significantly associated with obesity (*p* < 0.001), with higher percentages of obesity observed among those who ate out more frequently.

Table 4 presents the results of a study on the predictors of obesity among medical students at King Khalid University. The independent variables included age, sex, university grade, and dietary habits. Being a male was associated with higher odds of obesity (OR = 3.5, 95% CI [1.6–7.8], *p* = 0.002). Older age was associated with higher odds of obesity (OR = 1.8, 95% CI [1.1–3.0, *p* = 0.019]. Being in grade 2 (OR = 38.8, 95%CI [4.0–375.8], having grilled meat (OR = 0.4, 95%CI, [0.2–0.99], *p* = 0.048), using artificial sweeteners (yes) [OR = 0.20, 95% CI [0.08–0.72], *p* = 0.012], and drinking sparkling bottled water (OR 8.6, 95%CI [1.2–63.8], *p* = 0.034) were significantly associated with obesity. 

## 4. Discussion

Overweight and obesity are on an alarming upward trend in both developed and developing countries, affecting almost every aspect of social and economic life, regardless of age, sex, or ethnicity [18]. This study examined the dietary habits and prevalence of obesity among medical students at King Khalid University in Saudi Arabia, focusing on the prevalence of obesity and unhealthy eating habits.

### 4.1. The Study Main Findings

This study found a significant association between weight and eating habits. Among medical students, a large sector was underweighted and obese. The survey showed that many students ate three meals a day and snacked once a day. A lot of them liked full-fat milk and added sugar to their drinks. Many students ate out a few times a week. This study also found that obesity was associated with university grades and student gender. Males were more likely to be obese. There were differences between male and female students in how often they snacked, added sugar to drinks, ate out, cooked meat, and used sparkling bottled water. The main predictors of obesity were male, in the second year of university, eating grilled meat, using artificial sweeteners, and drinking sparkling water.

### 4.2. Interpretation of the Main Study Findings

Prevalence of obesity and overweight: In this study, we found that 21.9% of the students were overweight and 14.6% were obese. In the same vein, in a study involving 512 individuals aged 12–19 years living in the Aseer region, the prevalence of overweight and obesity was 33.6% and 20.5%, respectively [19]. Similarly, the prevalence of overweight, obesity, and high blood sugar among medical students at Qassim University in Saudi Arabia was as follows: 27.1% were overweight and 14% were obese [20]. In a study conducted by Alharbi et al. [21] at Qassim University, it was found that out of 405 medical students aged 19–25 years, 8.4% were classified as obese and 21.7% were overweight. It should be noted that a high prevalence of overweight and obesity has also been reported among non-Saudi youth. A cross-sectional survey conducted at four medical colleges in Lahore, Pakistan, focused on 244 medical students (85 males, 159 females) with a median age of 20 years. The study revealed that approximately 30.5% of males and 16% of females had a BMI ≥ 25.0 kg/m^2^ [22]. Similar results were found in India [23], Egypt [24], Syria [25], and Ghana [26]. Several key factors may explain the differences in the prevalence of obesity among medical students in these studies, such as changes in dietary habits and activity levels [27], environmental influences such as access to healthy food options and cultural norms around diet [28], body image [29], socioeconomic status [30], variations in health awareness and access to healthcare [31]. The findings highlight the importance of targeted health initiatives and education programs within educational institutions to raise awareness about the risks of obesity and promote healthier lifestyle choices, especially among medical students. It is critical that policymakers, healthcare professionals, and educators work together to develop effective strategies to combat the growing prevalence of overweight and obesity among students and young adults. Community-based interventions are one of the approaches that can be adopted. This approach has been proven to be effective in the management of many health conditions [32], including obesity [33].

Gender-based prevalence of obesity: We found that the prevalence of obesity and overweight was significantly higher among male students than among female students. Similar findings have been reported in many studies conducted in Saudi Arabia [34,35]. A study conducted by Alqarni [36] found that males exhibited a higher prevalence of overweight, while females show a higher prevalence of obesity. Understanding these gender-specific differences is critical to developing targeted interventions and public health initiatives to combat obesity and promote healthier lifestyles in students. Efforts to reduce obesity prevalence should consider tailored approaches that consider the unique challenges and determinants that influence weight status in specific gender populations. By addressing these disparities, we can help create a healthier and more equitable environment for all students.

Financial income and obesity: we did not find a significant association between income and the prevalence of obesity and overweight. In the same vein, a systematic review and meta-analysis explored the association between income and obesity, considering both social causation (lower income leading to higher obesity risk) and reverse causality (obesity causing lower income due to discrimination and stigmatization). An analysis of 21 studies suggested an association between lower income and subsequent obesity, although the significance decreased after adjustment for publication bias. Conversely, evidence for reverse causality was more consistent, indicating that obesity may influence subsequent income [37]. These findings highlight the complex interaction between socioeconomic factors and obesity, emphasizing the importance of nuanced approaches to understanding and addressing the underlying causes of weight-related health outcomes.

Our research of university grades revealed a statistically significant association between second-year students and obesity. Second-year students had higher odds ratios of becoming obese in the multivariate analysis, which could be attributable to differences in stress levels and study loads. Qalawa et al. [38] found strong associations between the body mass index of Saudi students and stress. Furthermore, Abdulghani et al. found that 63% of medical students experienced stress, with severe stress affecting 25% of the participants. Stress levels differed significantly between grades [39]. Thus, it is critical to establish focused intervention programs for second-year students, improved student support services, campus-wide health efforts, collaborative research and policy creation, and longitudinal studies to assess health outcomes over time. These measurements seek to improve student well-being and promote healthy lifestyles in university settings. 

### 4.3. Dietary Habits of the Studied Participants

Use of artificial sweeteners: we found that using artificial sweeteners was significantly associated with a 76% decrease in the odds of being obese. One meta-analysis, on the other hand, provided a rigorous evaluation of the scientific evidence available on low-calorie sugars (LCSs) and body weight and composition. However, data from a randomized control trial showed that substituting LCS options for their regular-calorie versions results in a modest weight loss and may be a useful dietary tool to improve compliance with weight loss or weight maintenance [40]. On the contrary, many studies have found a negative effect of using artificial sweeteners, with those using these products gaining more weight [41,42]. Pang et al. [43] reported an insignificant association between artificial sweeteners and weight gain and controlling blood glucose. Recently, the World Health Organization advised against using these products to reduce weight [44].

Eating outside homes (EOH): In this study, we found a significant association between EOH and the practice of dining away from home, commonly referred to as EOH, which has undergone significant evolution throughout the centuries. The introduction of the fast food concept marked a pivotal moment in this evolution. In contemporary times, various dining concepts such as bars, restaurants, lunch counters, snack bars, and buffets contribute to the diversity of EOH experiences [45]. The food consumed during dining out experiences usually tend to have a high energy content. This characteristic can play a significant role in contributing to an excessive general energy intake, potentially leading to obesity [46]. To mitigate the negative impact of EOH, it is essential to implement nutritional education programs, enforce clear menu labeling, promote healthy eating environments by collaborating with food service providers, engage local communities through workshops, and implement regulatory measures to curb impulse consumption and encourage healthier dietary patterns.

Sparkling water, also known as soda water, undergoes a process in which water is pressurized with carbon dioxide to produce effervescence. To put it differently, water is infused with pressurized carbon dioxide, resulting in those delightful tiny bubbles. There is a scarcity of research on the association between sparkling water and obesity. In this study, we observed that the consumption of sparkling water was associated with an eightfold increase in the likelihood of obesity. An animal study conducted by Eweis et al. [47] observed that subjects consuming gaseous beverages over the course of approximately one year exhibited accelerated weight gain compared to the control group, that were provided with regular degassed carbonated beverages or tap water. This observed phenomenon was related to elevated levels of the hunger hormone ghrelin, which contributed to increased food intake.

#### 4.3.1. Implications of the Study Findings

This study highlights the need for comprehensive health education programs for medical students, addressing nutritional aspects, physical activity, stress management, and financial support. It also emphasizes the need for gender-specific health initiatives and targeted interventions for different grade levels. Institutional policies should promote healthy eating habits and physical activity. Collaborative efforts between educational institutions and healthcare providers are crucial for regular health check-ups, nutritional counseling, and mental health support.

#### 4.3.2. Strengths and Limitations

This study demonstrates several strengths, primarily stemming from its comprehensive observational cross-sectional design, which enables a thorough examination of the factors impacting BMI among medical students. Face-to-face interviews are an important asset, as they improve data quality by capturing detailed information on the eating habits of the participants. The involvement of trained interviewers ensures the internal validity of the study findings. Additionally, pilot testing contributes to the refinement of the questionnaire. However, this study also faces certain limitations. Its cross-sectional nature restricts the ability to draw causal inferences about the observed associations. Furthermore, reliance on self-reported data introduces the potential for bias. Lastly, the study’s focus on a single center may limit the generalizability of its findings beyond the specific study population.

## 5. Conclusions

This study found that large sectors of the studied students were classified as underweight, overweight, or obese, emphasizing the importance of addressing weight-related health concerns. Moreover, this study discovered significant associations between obesity and a variety of demographic factors, including age, gender, and university grade. Furthermore, dietary habits such as eating grilled meat, adding salt, drinking sparkling water, and using artificial sweeteners were found to be significantly associated with obesity. Finally, the frequency of eating out was identified as a significant risk factor for obesity. These findings emphasize the multifaceted nature of obesity and the importance of considering a variety of lifestyle factors when understanding and addressing its prevalence. Consequently, medical students should be educated on maintaining a healthy weight, balanced nutrition, and regular physical activity. Nutritional counseling services should be offered to guide students in making informed choices.

## Figures and Tables

**Table 1:** Demographic profile of medical students from King Khalid University (n = 540).

Variables	Level	N	%
Age category	18–19 years	133	24.6
	20–22 years	234	43.3
	>22 years	173	32.0
	Mean ± Sd	21.7 ± 1.5	
Sex	Male	288	53.3
	Female	252	46.7
Academic Year	2nd grade	115	21.3
	3rd grade	134	24.8
	4the grade	112	20.7
	5th grade	122	22.6
	6th grade	57	10.6
Income level	Below Average	7	1.3
	Average	443	82.0
	Above Average	89	16.5
Household	Below 3	53	9.8
	3–6 members	208	38.5
	>6 members	279	51.7
	Mean ± Sd	6.6 ± 2.8	

Sd: standard deviation.

**Table 2:** Differences in responses between male and female medical students at King Khalid University (N = 540).

Dietary Habits		Total		Sex				*p*
				Male		Female		
		n	%	n	%	n	%	
How many meals do you usually consume daily?	1 Meal	32	5.9	17	5.9	15	6.0	0.922
	2 Meals	234	43.3	128	44.4	106	42.1	
	3 Meals	221	40.9	117	40.6	104	41.3	
	4 Meals	41	7.6	21	7.3	20	7.9	
	5 or more meals	12	2.2	5	1.7	7	2.8	
Do you consume meals at regular times?	No	213	39.4	113	39.2	100	39.7	0.990
	Yes, but only some of them	254	47	136	47.2	118	46.8	
	Yes, all of them	73	13.5	39	13.5	34	13.5	
How often do you snack between the meals?	Never	23	4.3	17	5.9	6	2.4	0.001
	1–3 times a month	48	8.9	29	10.1	19	7.5	
	Once a week	69	12.8	43	14.9	26	10.3	
	Few times a week	143	26.5	92	31.9	51	20.2	
	Once a day	151	28	70	24.3	81	32.1	
	Few times a day	106	19.6	37	12.8	69	27.4	
What type of milk and dairy beverages you usually consume?	Full fat	340	63	192	66.7	148	58.7	0.065
	Low fat	177	32.8	88	30.6	89	35.3	
	No fat (Skimmed)	23	4.3	8	2.8	15	6.0	
Do you add any sugar to your hot beverages, e.g., tea, hot chocolate, coffee	No	123	22.8	66	22.9	57	22.6	0.001
	Yes, I add one teaspoon of sugar (or honey)	213	39.4	95	33.0	118	46.8	
	Yes, I add two or more teaspoons of sugar (or honey)	162	30	108	37.5	54	21.4	
	Yes, I use artificial sweeteners (low-caloric substitute for sugar)	42	7.8	19	6.6	23	9.1	
Do you add salt to your meals and sandwiches once prepared?	No	118	21.9	59	20.5	59	23.4	0.683
	Yes, but only sometimes	187	34.6	103	35.8	84	33.3	
	Yes, I add salt to most of my meals	235	43.5	126	43.8	109	43.3	
How often do you eat out, e.g., in a restaurant, café, canteen?	Never	20	3.7	12	4.2	8	3.2	0.001
	1–3 times a month	119	22	37	12.8	82	32.5	
	Once a week	139	25.7	54	18.8	85	33.7	
	Few times a week	184	34.1	121	42.0	63	25.0	
	Once a day	54	10	44	15.3	10	4.0	
	Few times a day	24	4.4	20	6.9	4	1.6	
Type of meat (fried)	No	465	86.1	259	89.9	206	81.7	0.006
	Yes	75	13.9	29	10.1	46	18.3	
Type of meat (roasted)	No	351	65	208	72.2	143	56.7	0.001
	Yes	189	35	80	27.8	109	43.3	
Type of meat (grilled)	No	351	65	208	72.2	143	56.7	0.001
	Yes	189	35	80	27.8	109	43.3	
Type of meat (stewed)	No	417	77.2	225	78.1	192	76.2	0.593
	Yes	123	22.8	63	21.9	60	23.8	
Type of meat (boiled)	No	389	72	224	77.8	165	65.5	0.001
	Yes	151	28	64	22.2	87	34.5	
Drinking regular boiled water	No	288	53.3	106	36.8	182	72.2	0.001
	Yes	252	46.7	58	20.1	194	77.0	

**Table 3:** Association between obesity and different participants characteristics.

Studied Variables		Non-Obese		Obese		*p*
		n	%	n	%	
Age (mean ± sd)		21.7 ±1.5		21.6 ± 1.6		0.724
Sex	Male	225	78.1	63	21.9	<0.001
	Female	236	93.7	16	6.3	
Grade	2nd grade	86	74.8	29	25.2	<0.001
	3rd grade	125	93.3	9	6.7	
	4the grade	96	85.7	16	14.3	
	5th grade	108	88.5	14	11.5	
	6th grade	46	80.7	11	19.3	
House holds	Below 3	46	86.8	7	13.2	0.952
	3–6 members	177	85.1	31	14.9	
	>6 members	238	85.3	41	14.7	
How would you describe your financial situation?	Below Average	7	100	0	0	0.358
	Average	380	85.8	63	14.2	
	Above Average	73	82	16	18	
How many meals do you usually consume daily?	1 Meal	28	87.5	4	12.5	0.693
	2 Meals	196	83.8	38	16.2	
	3 Meals	192	86.9	29	13.1	
	4 Meals	36	87.8	5	12.2	
	5 or more meals	9	75	3	25	
Do you consume meals at regular times?	No	179	84	34	16	0.421
	Yes, but only some of them	222	87.4	32	12.6	
	Yes, all of them	60	82.2	13	17.8	
How often do you snack between the meals?	Never	22	95.7	1	4.3	0.444
	1–3 times a month	43	89.6	5	10.4	
	Once a week	61	88.4	8	11.6	
	Few times a week	121	84.6	22	15.4	
	Once a day	128	84.8	23	15.2	
	Few times a day	86	81.1	20	18.9	
Do you add any sugar to your hot beverages, e.g., tea, hot chocolate, coffee	No	143	86.7	22	13.3	0.081
	Yes, I add one teaspoon of sugar (or honey)	188	88.3	25	11.7	
	Yes, I add two or more teaspoons of sugar (or honey)	130	80.2	32	19.8	
Use artificial sweeteners	No	430	86.3	68	13.7	0.027
	Yes	31	73.8	11	26.2	
Do you add salt to your meals and sandwiches once prepared?	No	103	87.3	15	12.7	0.028
	Yes, but only sometimes	168	89.8	19	10.2	
	Yes, I add salt to most of my meals	190	80.9	45	19.1	
Sparkling bottled water	No	419	84.5	77	15.5	0.048
	Yes	42	95.5	2	4.5	
How often do you eat out, e.g., in a restaurant, café, canteen?	Never	19	95	1	5	<0.001
	1–3 times a month	107	89.9	12	10.1	
	Once a week	129	92.8	10	7.2	
	Few times a week	150	81.5	34	18.5	
	Once a day	40	74.1	14	25.9	
	Few times a day	16	66.7	8	33.3	

**Table 4:** Predictors of obesity among medical students in King Khalid University (N = 540).

Studied Variables	B	S.E.	*p*	OR	95% CI	
					LCI	UCI
Constant	−18.1	6.8	0.008	0.0		
Sex (male)	1.2	0.4	0.002	3.5	1.6	7.8
Age (in year)	0.6	0.3	0.019	1.8	1.1	3.0
University grade			0.0001			
Grade 6 (ref)						
Grade 2	3.7	1.2	0.002	38.8	4.0	375.8
Grade 3	0.9	0.9	0.287	2.5	0.5	14.2
Grade 4	1.3	0.7	0.055	3.6	1.0	13.6
Grade 5	0.7	0.6	0.219	2.0	0.7	5.9
Having regular meals (yes)	0.05	0.3	0.877	1.0	0.6	1.8
What type of milk and dairy beverages you usually consume?			0.667			
No fat (Skimmed) (ref)						
Full fat	1.0	1.2	0.396	2.7	0.3	25.3
Low fat	1.0	1.2	0.369	2.8	0.3	27.4
Type of meat (grilled)	−0.8	0.4	0.048	0.42	0.20	0.99
Type of meat (roasted)	0.1	0.4	0.783	1.1	0.6	2.2
Type of meat (stewed)	−0.5	0.4	0.128	0.6	0.3	1.2
Type of meat (boiled)	0.4	0.4	0.326	1.5	0.7	3.1
Do you add any sugar to your hot beverages, e.g., tea, hot chocolate, coffee			0.329			
Yes, I add two or more teaspoons of sugar (or honey) (ref)						
No	−0.6	0.4	0.169	0.6	0.2	1.3
Yes, I add one teaspoon of sugar (or honey)	−0.4	0.3	0.276	0.7	0.3	1.4
Do you add salt to your meals and sandwiches once prepared?			0.082			
Yes, I add salt to most of my meals (ref)						
No	−0.2	0.4	0.576	0.8	0.4	1.7
Yes, but only sometimes	−0.7	0.3	0.025	0.5	0.3	0.9
Type of drinking water (regular boiled)	1.1	0.7	0.122	2.9	0.7	11.6
Type of drinking water (desalinated water)	0.5	0.7	0.489	1.6	0.4	6.1
Type of drinking water (sparkling bottled water)	2.2	1.0	0.034	8.6	1.2	63.8
Type of drinking water (flavored bottled water)	0.7	0.9	0.441	2.0	0.4	10.7
How often do you eat out, e.g., in a restaurant, café, canteen?			0.261			
Few times a day (ref)						
Never	−1.9	1.2	0.122	0.2	0.01	1.6
1–3 times a month	−1.0	0.6	0.127	0.4	0.1	1.3
Once a week	−1.3	0.7	0.038	0.3	0.1	0.9
Few times a week	−0.6	0.6	0.290	0.6	0.2	1.7
Once a day	−0.6	0.6	0.373	0.6	0.2	2.0
Snacks (yes)	0.2	0.3	0.491	1.2	0.7	2.2
Artificial sweeteners (yes)	−1.4	0.6	0.012	0.2	0.1	0.7

Dependent variable (obesity).

## Data Availability

Data are available upon request by emailing the corresponding authors.
